# A multifactorial intervention to improve cardiovascular outcomes in adults with type 2 diabetes and current or previous diabetic foot ulcer disease: Protocol for a multi‐centre randomised control trial (MiFoot study)

**DOI:** 10.1111/dme.70028

**Published:** 2025-04-05

**Authors:** Tolu Onuwe, Patrick J. Highton, David Batchelor, Alan Brennan, Molly Caba, Melanie J. Davies, Mark P. Funnell, Frances Game, Clare L. Gillies, Agnieszka Glab, Laura J. Gray, Edward Gregg, Michelle Hadjiconstantinou, Vicky Hall, Vicki Johnson, John R. Petrie, Dan Pollard, Hannah Rowntree, Solomon Tesfaye, Jonathan Valabhji, David Webb, Francesco Zaccardi, Kamlesh Khunti

**Affiliations:** ^1^ Diabetes Research Centre, Leicester General Hospital University of Leicester Leicester UK; ^2^ National Institute for Health and Care Research Applied Research Collaboration East Midlands Leicester UK; ^3^ Patient and Public Involvement and Engagement Representative Leicester UK; ^4^ School of Medicine and Population Health University of Sheffield Sheffield UK; ^5^ National Institute for Health and Care Research Leicester Biomedical Research Centre Leicester UK; ^6^ Department of Research and Development University Hospitals of Derby and Burton NHS Foundation Trust Derby UK; ^7^ Leicester Real World Evidence Unit, Leicester Diabetes Centre University of Leicester Leicester UK; ^8^ Leicester Diabetes Centre University Hospitals of Leicester NHS Trust, Leicester General Hospital Leicester UK; ^9^ Department of Population Health Sciences University of Leicester Leicester UK; ^10^ Leicester British Heart Foundation Centre of Research Excellence University of Leicester Leicester UK; ^11^ School of Population Health RCSI University of Medicine and Health Sciences Dublin Ireland; ^12^ School of Public Health Imperial College London London UK; ^13^ School of Health and Wellbeing, College of Medical Veterinary and Life Sciences University of Glasgow Glasgow UK; ^14^ Diabetes Research Unit Sheffield Teaching Hospitals NHS Foundation Trust Sheffield UK; ^15^ Department of Metabolism, Digestion and Reproduction, Faculty of Medicine, Chelsea and Westminster Hospital Campus Imperial College London London UK

**Keywords:** cardiovascular disease, diabetes‐related foot ulcer disease, digital‐based programme, lifestyle, mortality, physical activity, self management education programme

## Abstract

**Background:**

In the United Kingdom, the prevalence of diabetes‐related foot ulcer disease (DFUD) is 6.3%, and cardiovascular disease (CVD) is the leading cause of mortality in people with DFUD. This study aims to evaluate the effectiveness of a multifactorial intervention to reduce CVD events and mortality in adults with type 2 diabetes (T2D) and DFUD.

**Methods:**

The MiFoot study is a multi‐centre, pragmatic randomised controlled trial to test intervention effectiveness and cost‐effectiveness compared to usual care that will include an internal feasibility study and a process evaluation. English‐speaking adults (≥18 years; *n* = 392) with T2D and current/previous (within 5 years) DFUD will be recruited from multiple sites across the United Kingdom and randomised 1:1 to intervention (MiFoot multifactorial intervention plus usual care) or control (usual care), with data collected at baseline, 12‐ and 24‐month follow‐up. The MiFoot intervention comprises an individualised assessment with a healthcare practitioner to optimise treatment and assess the suitability of physical activity participation; group‐based disease self management education and physical activity sessions; and a digital‐based programme, consisting of cohort‐relevant topics, physical activity guidance and peer support functionality. The primary outcome will be extended major adverse cardiovascular events (MACE, i.e. myocardial infarction, stroke, cardiovascular death, peripheral arterial bypass, coronary artery bypass, coronary angioplasty or peripheral artery angioplasty) at 24 months.

**DISCUSSION:**

This study will provide evidence on the feasibility and clinical effectiveness, and cost‐effectiveness of a multifactorial intervention to prevent or slow the progression of CVD‐related complications in the extremely high‐risk population with T2D and DFUD.

## 1 INTRODUCTION

Diabetes‐related foot ulcer disease (DFUD), defined as an ulcer below the malleoli, is a devastating complication of diabetes and is associated with significantly increased risks of cardiovascular disease (CVD) and premature mortality.^1^ In the United Kingdom, the prevalence of DFUD is 6.3%,^2^ and prevalence and disease burden are substantially higher in people with Type 2 (T2D) than Type 1 (T1D)^3^ diabetes. Five‐year mortality in individuals with DFUD is 40%,^4^ which is worse than many cancers; yet DFUD is consistently perceived as less life‐threatening.^5^ Moreover, the financial burden of DFUD on the NHS is higher than that of breast, prostate and lung cancer combined.^6^

While CVD is the leading cause of death in people with DFUD,^7^ research conducted in people with DFUD typically focuses on ulcer prevention and management.^8^ Although imposed physical inactivity and immobilisation with other long‐term conditions in DFUD drive CVD risks,^9^, ^10^ they do not entirely account for the excess CVD burden seen in this population. Accordingly, research into aggressive CVD risk modification in people with DFUD is of clinical relevance.

There is evidence of improved CVD risk from structured self management education and multiple risk factor control in people with T2D and microalbuminuria.^11^ However, these interventions have not been widely adopted for people with T2D and DFUD. Re‐designing previous high‐quality trials in T2D patients without DFUD specifically for the needs of the DFUD patient groups, for example, by replacing leg load‐bearing physical activity with arm ergometer exercises,^5^ provides an opportunity for clinical benefit in this high‐risk cohort. While there is limited evidence supporting non‐load‐bearing exercises in people with DFUD, there exists some literature on the efficacy of non‐load bearing exercises, either within research or as routine care, and philosophy‐backed behavioural interventions on CVD risks in other chronic diseases.^9^, ^10^, ^12^, ^13^ Therefore, these types of exercises provide opportunities for those with DFUD to gain the cardiovascular benefits of exercise without the risk of further foot problems.^14^ Providing offloading of foot and ankle joints does not have to exclude providing safe, effective, and protective vascular exercises like seated upper body cardiovascular exercise programmes which may be beneficial for people with active foot disease.^15^ Hence, this trial will investigate the clinical and cost effectiveness of a multifactorial complex intervention to prevent or slow the development of CVD‐related complications in people with T2D and DFUD, compared to usual care.

## 2 METHODS

### 2.1 Study design

**FIGURE 1:**
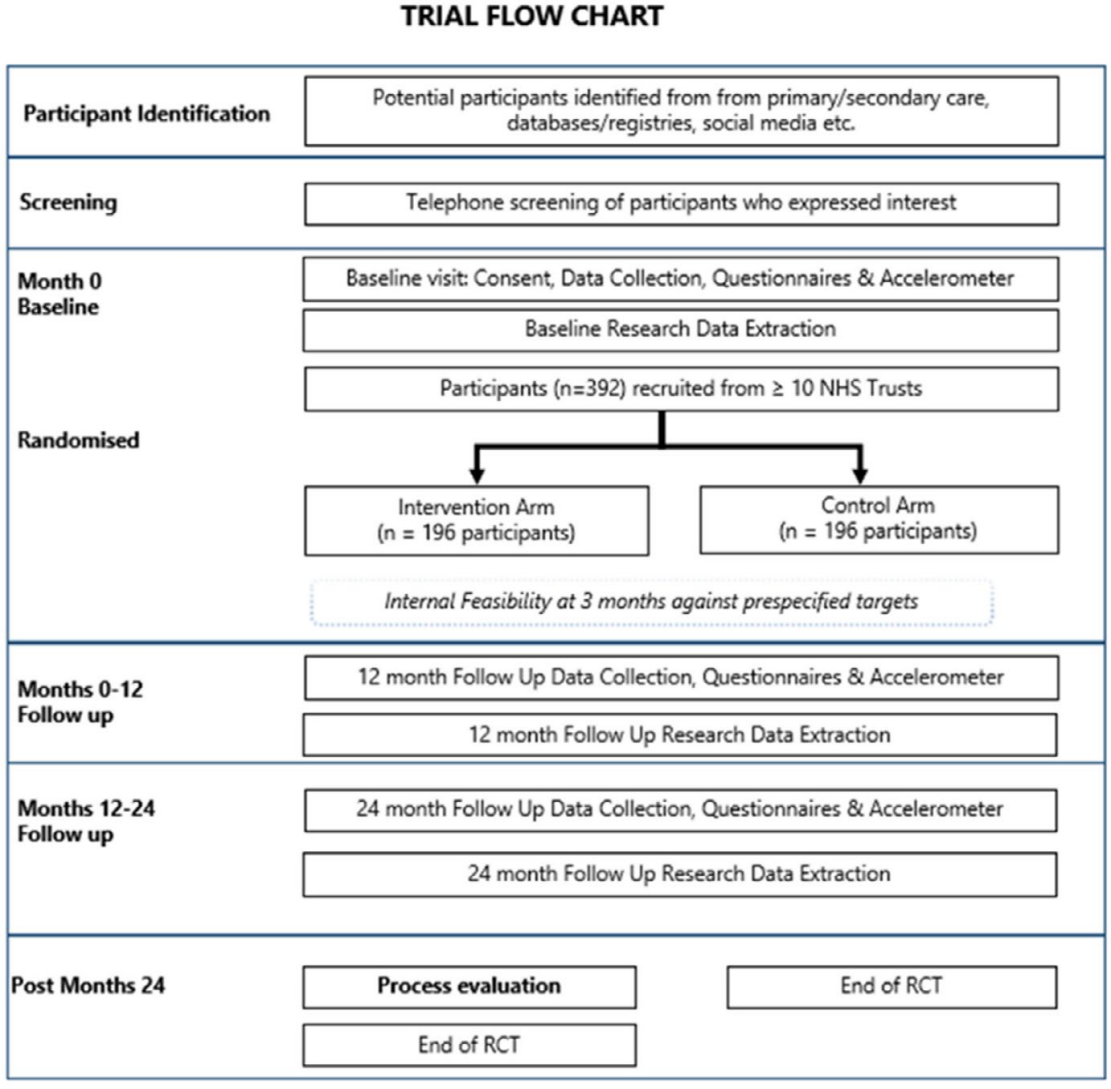
Schematic representation of the processes of the study.

The MiFoot study is a multi‐centre, pragmatic randomised controlled trial (RCT) designed to test the clinical and cost‐effectiveness of the MiFoot intervention compared to usual care, with an internal feasibility study and process evaluation. This study was granted a favourable ethical opinion by the North East–York Research Ethics Committee (23/NE/0136), received Health Research Authority approval, and has been prospectively registered on the Clinical Trials Registry (https://www.isrctn.com/ISRCTN13413505). Figure 1 describes participants progression through the study.

### 2.2 Aims

Primary aim: Compare the MiFoot intervention with usual care for the prevention of CVD events in people with T2D and current or previous DFUD.

Secondary aims include: (1) Estimate the cost‐effectiveness of the MiFoot programme in a real‐world clinical setting; (2) Evaluate the sustainability of the programme by completing a process evaluation; and (3) Monitor the recruitment and stop‐go criteria with an internal feasibility study.

### 2.3 Study population

#### 2.3.1 Inclusion and exclusion criteria

The study will include adults diagnosed with T2D and with current or previous (within the past 5 years) DFUD. Participants must understand English, be able and willing to fulfil the study requirements and not currently be taking part in a clinical trial for an investigational medicinal product or any other disease management or lifestyle‐related intervention trial. Patients diagnosed with other forms of diabetes and forms of ulceration not attributable to peripheral neuropathy or peripheral vascular disease will be excluded, as well as patients with serious illnesses or events associated with a life expectancy of <1 year. Patients unable to provide consent, with a planned major surgery, or requiring renal replacement therapy, who are currently pregnant or actively trying to conceive, will be excluded.

#### 2.3.2 Participant identification

Potential participants will be identified via primary and secondary care sites supported by Regional Research Delivery Networks, existing participants on volunteer databases, community organisations and networks, and other avenues including social media. We will leverage equality, diversity and inclusion (EDI) guidelines while promoting, identifying and inviting participants to the study, and will collect EDI‐relevant data (e.g. sociodemographic data).

### 2.4 Trial procedures

#### 2.4.1 Informed consent

Following eligibility confirmation, participants will be given the opportunity to ask any questions relating to their study involvement and will give informed consent to a healthcare professional (HCP).

#### 2.4.2 Baseline measure data collection

Data collected will include participants' age, sex, ethnicity, height, weight, resting heart rate, blood pressure and DFUD severity score based on ulcer Site–Ischemia–Neuropathy–Bacterial‐infection–Area–and Depth (SINBAD), if the participant has an active foot ulcer.

#### 2.4.3 Baseline research data extraction

Routine health data, medical history, all current diagnoses and dates, procedures/surgeries and medications, will be extracted from participants' medical records. Routine biomarker results, as listed in Table 1, that are within 6 months, will be extracted or requested through the GP.

**TABLE 1:** Secondary outcomes, measures and time of measurement during the study.

Secondary outcomes	Measures	Visits		
		Baseline visit, Month 0	Remote, 12‐month follow‐up	Remote, 24‐month follow‐up
Health outcomes	Composite renal end points: End‐stage kidney disease (defined as dialysis, transplantation, or a sustained (>3 months) estimated Glomerular Filtration Rate [eGFR] of <15 mL/min/1.73 m^2^) Doubling of the serum creatinine level Or death from renal causes 3‐Point MACE (non‐fatal myocardial infarction, non‐fatal stroke and cardiovascular death) and components of the extended MACE All‐cause mortality Lower limb major amputation Self reported re‐ulceration		X	X
Patient‐reported outcomes	Using Questionnaires: Distress using Problem Area in Diabetes‐20 (PAID‐20) Self‐efficacy using Diabetes Management Self‐Efficacy Scale‐15 (DMSES‐15) Quality of Life using Diabetes Foot Ulcer Disease (Diabetes Foot Scale‐Short Form [DFS‐SF], generic EuroQoL‐5Dimension‐5Levels [EQ‐5D‐5L]) Depression and anxiety using Hospital Anxiety and Depression Scale (HADS) Health resource use, such as primary care visits, emergency department visits, hospitalisations and medication use using Modular Resource‐Use Measure (ModRUM) Medication adherence using the Morisky Medication Adherence Scale (MMAS‐8) Diet using the Short Form Food Frequency Questionnaire Sleep duration/quality and physical activity volume/intensity measured objectively using wrist worn accelerometers	X	X	X
Biomedical markers	Blood pressure (systolic, diastolic and heart rate) (mmHg, BPM) Low‐density lipoprotein‐cholesterol (LDL‐C) (mmol/L) High‐density lipoprotein‐cholesterol (HDL‐C) (mmol/L) Total cholesterol (TC) (mmol/L) Triglycerides (TG) (mmol/L) Glycated haemoglobin (HbA1c) (% and mmol/mol) Estimated Glomerular Filtration Rate (eGFR) (mL/min/1.73 m2) Urine albumin: creatinine ratio (uACR).	X	X	X
Anthropometric	Weight Body mass index (BMI)	X	X	X
Demographic variables (collected for exploratory stratified analyses)	Age Sex Ethnicity T2DM duration DFUD duration Socio‐economic score (Index of Multiple Deprivation (IMD): a postcode‐based measure of socio‐economic score) Medications (glucose‐lowering, lipid‐lowering, blood pressure‐lowering, antiplatelet and antidepressants)	X	X	X
CGM Metrics	Time in range 3.9‐10 mmol/L Time above range (>10 mmol/L) Time below range (<3.9 mmol/L) sensor usage data (proportion of time the sensor is used)	X	X	X

#### 2.4.4 Accelerometer

Participants will wear an accelerometer on their non‐dominant arm for 8 days to capture habitual activities, sleep duration and quality, overall physical activity volume and intensity profile, along with time spent sedentary, in light‐intensity physical activity, and in moderate‐to‐vigorous physical activities.

#### 2.4.5 Continuous glucose monitors (CGMs)

All participants will be offered CGM devices (Freestyle Libre 2) to ensure trial results are obtained in the context of optimised usual care. CGMs will be optional; we will perform exploratory analyses of sensor glucose levels. Data will be downloaded remotely via LibreLink.

#### 2.4.6 Questionnaires

Participants will complete the following questionnaires at baseline, 12 and 24 months (Table 1):

- EuroQol 5‐Dimension 5‐Level (EQ‐5D‐5L): Measures health‐related quality of life (QoL) specifically in chronic health conditions.
- Hospital Anxiety and Depression Scale (HADS): Measures the severity of symptoms of anxiety and depression.
- ModRUM: Includes questions on healthcare resource use covering emergency care, outpatient care, GP visits (including home‐visit or remotely accessed) and NHS HCP visits.
- Diabetic Foot Ulcer Scale–Short Form (DFS‐SF): Assesses the impact of foot ulcers and their treatment on the QoL of people with diabetes.
- Diabetes Self Management Efficacy Scale (DMSES‐15): Assesses respondents' confidence levels in their blood sugar, diet and level of exercise management.
- Problem Areas in Diabetes Questionnaire (PAID‐20): Measures diabetes stress.
- Morisky Medication Adherence Scale (MMAs‐8): Measures medication‐taking behaviour.
- Diet (short‐form food frequency questionnaire): Assesses dietary habits.

#### 2.4.7 Randomisation

Participants will be randomly allocated 1:1 to the control or intervention arm using an online 24‐h system provided by the Derby Clinical Trials Support Unit (DCTSU). Block randomisation will be stratified by site, sex (men; women) and age (<50; ≥50 years).

#### 2.4.8 Blinding

Given the design of the intervention, the study team and participants will not be blinded to the intervention. The study statistician is masked, but no code‐break or emergency unblinding will be required. To reduce bias, the primary and most of the secondary outcomes will be collected from the participants' medical records rather than specifically collected for the study. The patient‐reported secondary outcomes will be collected using validated questionnaires. As such, there are no outcome assessors involved in the study.

### 2.5 Intervention

#### 2.5.1 The MiFoot programme

#### 2.5.2 Public and patient involvement and engagement (PPIE)

PPIE members informed the design of the MiFoot intervention and RCT, including reviewing patient‐facing documentation, lay summaries and dissemination strategies. PPIE members will be proactively involved throughout the trial to support participant recruitment, data collection, results interpretation and dissemination. A PPIE member also sits on the Trial Steering Committee.

The MiFoot programme is a complex, multifactorial, evidence‐based, theory‐driven, person‐centred intervention designed to improve cardiovascular health and fitness and comprises three components:

1. An individualised 1:1 appointment with an HCP that focuses on medical management and intensive multifactorial CVD risk‐factor control like glycated haemoglobin (HbA1c), blood pressure, body mass index (BMI), anxiety, depression and lipids. An evidence‐based, pictorial health profile is used to plot baseline measurements and is used to help participants understand their health measurements, facilitate discussions and a formulate an agreed treatment plan. Participants' medical history will be reviewed, contraindications to exercise screened for and advice regarding weight‐bearing status for those with active foot ulcer disease as well as the intensity of physical activity (light or moderate) will be carried out during this appointment. An ECG will also be carried out. This appointment will also help address clinical inertia.
2. Group‐based disease self management education and physical activity sessions, comprising education regarding disease knowledge, risk‐factor control, diet and weight management, physical activity, emotional management and goal setting. Sessions will include opportunities for physical activity instruction, participation and supervision. Participants' heart rate and rated perceived exertion will be monitored during the physical activity sessions to ensure an appropriate response to activity as well as that the intensity of activity is not exceeded. These sessions are delivered by two trained facilitators, at least one of whom is an HCP.
3. A bespoke DFUD‐related digital programme, based on the pre‐existing MyDesmond platform for the management of T2D, to supplement the group‐based sessions, comprises relevant learning sessions, physical activity guidance, health trackers, ask‐the‐expert functionality and forums for peer support.

**FIGURE 2:**
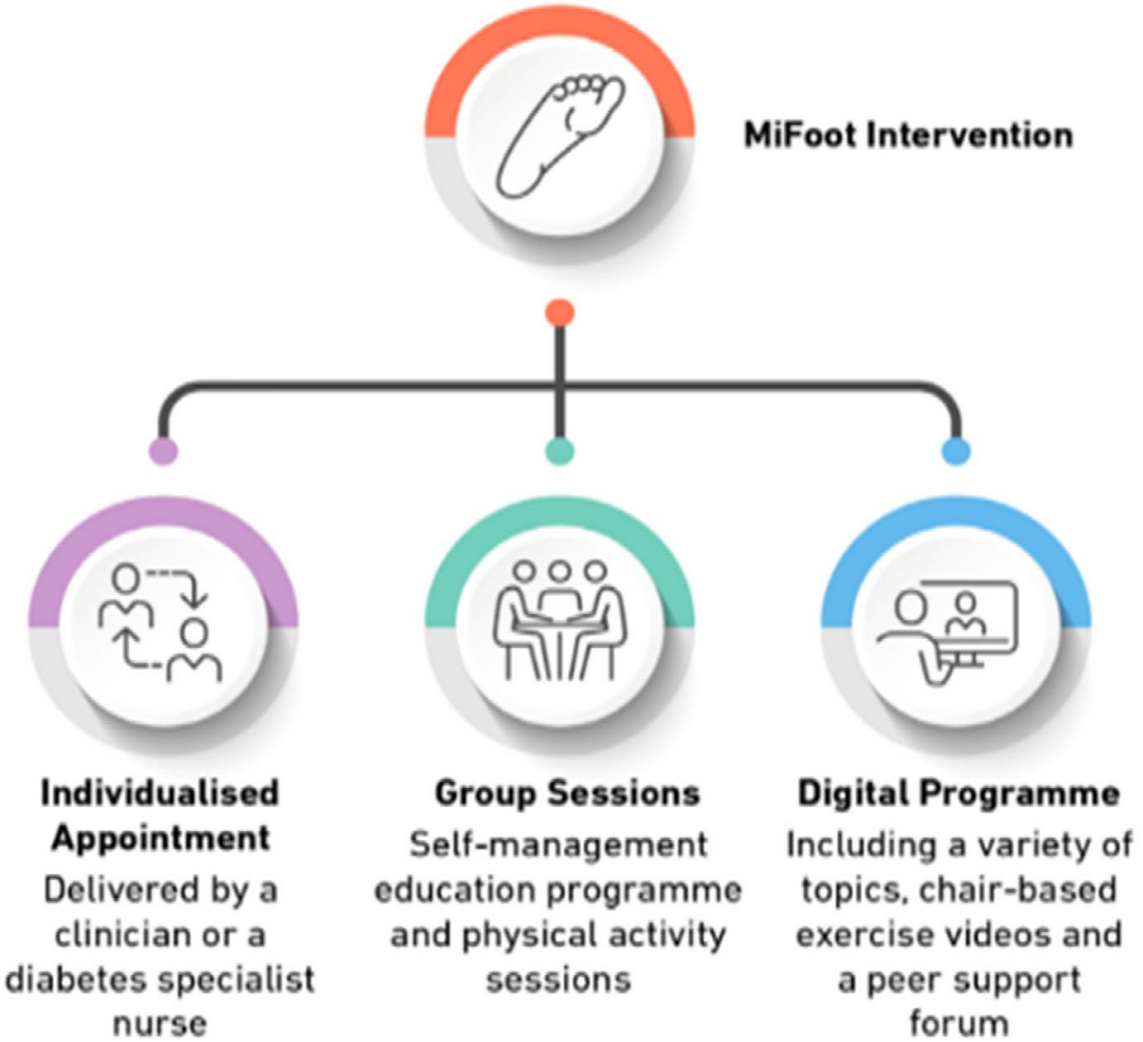
Components of the MiFoot Intervention.

The intervention components are displayed in Figure 2. The intervention development process will be further detailed in a future publication.

Prior to participation in the physical activity component of the intervention, participants will undergo precautionary screening to ensure safety. This will include an electrocardiogram (ECG) to identify any potential undiagnosed cardiac problems, assessment of any active ulcers and completion of a specifically designed assessment form to safely determine participants' readiness and ability for physical activity.

## 3 Outcomes

The primary outcome is the incidence of extended Major Adverse Cardiovascular Events (MACE) at 24 months, identified using routine healthcare data. The extended MACE definition for MiFoot is context‐specific and is defined as myocardial infarction, stroke, cardiovascular death, peripheral arterial bypass, coronary artery bypass, coronary angioplasty or peripheral artery angioplasty.^16^ The secondary outcomes are health, patient‐reported, biomedical and anthropometric outcomes, described in Table 1.

### 3.1 Follow‐up assessments

Participants will be followed up for 24 months using the same measures as the baseline visit through questionnaires, accelerometers, routine health data and health outcomes (Tables 1 and 2). There will be no on‐site visits for the 12‐ and 24‐month assessments.

**TABLE 2:** Schedule of procedures.

Procedures	Visits			
	Pre‐screening	Baseline visit, Month 0	Remote, 12‐month follow‐up	Remote, 24‐month follow‐up
Invitation and EOI	XX			
Telephone screening	XX			
Informed consent		XX		
Face to face eligibility screening		XX		
Demographics		XX		
Baseline Research Data Extraction		XX		
SINBAD Score		XX		
Consent		XX		
Inform GP of study participation		XX		
Randomisation		XX		
Anthropometric		XX	XX	XX
Patient‐reported outcomes Questionnaires		XX	XX (±4 weeks)	XX (±4 weeks)
Accelerometer		XX (±4 weeks)	XX (±4 weeks)	XX (±4 weeks)
CGM provision		XX^a^		
ECG		X		
Health outcomes data collection		XX	XX	XX
Biomedical markers		XX	XX	XX
Physical activity screening		X		
Adverse event assessments Safety measures		X	X	X
Process evaluation observations		X		
Process evaluation feedback surveys		X^b^		
Process evaluation questionnaires			X	
Process evaluation interviews				After 24th month

*Note*: X: Intervention arm only; XX: All study participants.

Abbreviations: ECG, electrocardiogram; EOI, expression of interest; GP, general practitioner; SINBAD, ulcer site, ischemia, neuropathy, bacterial infection, area and depth.

^a^ All participants will be offered CGM devices at their baseline appointment; though if they decline at this stage, they can change their mind throughout the trial.

^b^ After each one‐to‐one and group sessions and 4 weeks after the digital programme, collected up to month 9 of the intervention delivery.

## 4 Sample size calculation

Three hundred and ninety‐two participants will be recruited across all sites. This calculation is based on detecting a 20% improvement in the survival rate for extended MACE (a much smaller reduction than a similar intervention in another high risk T2D population^17^) at 24 months from 65.6% (34.4% experience an event) to 78.7% (21.3% experience an event). The 65.6% rate in the control arm at 24 months was extrapolated from two of our previous studies in this population.^11^, ^18^ Assuming 80% power and 5% alpha, a sample size of 372 participants is required. Allowing for a 5% drop out (low because primary outcome will be collected by data linkage) similar to previous work,^14^ the number of participants required increases to 392. The sample size calculation may be proactively refined if any further relevant data emerge in the literature during the study.

## 5 Secondary study aims

### 5.1 Cost effectiveness of MiFoot

Two health economic analyses will be conducted to assess the cost‐effectiveness of MiFoot: (1) an analysis of economic data collected within the trial; and (2) a long‐term economic modelling analysis (lifetime horizon). Both analyses will take an NHS and personal social services perspective, and the long‐term analysis will discount future costs and quality‐adjusted life years (QALYs) at 3.5% per annum, based on National Institute for Health and Care Excellence (NICE) recommendations.^19^ Both analyses will estimate incremental cost‐effectiveness ratios for study outcomes, including QALYs, comparing the MiFoot intervention to usual care. The long‐term model will be based on our existing health economic model, the School for Public Health Research Type 2 Diabetes Treatment Model (SPHR‐T2DMT).^20^

## 6 Sustainability of MiFoot

### 6.1 Internal feasibility

An internal feasibility study, designed to evaluate the viability of the study during its initial phases, will be undertaken. This will evaluate the feasibility of recruiting to target in the study. Hence, the stop‐go criteria are based on the first 3 months of recruitment, when recruitment should be 25% complete (i.e. 98 participants). To allow for a slower start, feasibility criteria will be set at 20% of the recruitment target (i.e. 78 participants). Based on the actual number of patients recruited after 3 months compared with the target of 78 participants (red: ≤49; amber: 50–77; green: ≥78), the Data Monitoring and Safety Committee (DMSC) will make a recommendation on how to proceed.

### 6.2 Adherence and retention

In order to increase participant engagement by tailoring the intervention to the individual, the different components of the MiFoot intervention are made optional, enabling participants to choose which component or combination of components they want to engage with. The intervention is also theoretically underpinned to support behaviour change and engagement throughout the trial. The physical and psychological challenges of this population were considered during the development phase, and the programme is tailored to address their needs. For example, to encourage adherence to the digital programme, we not only have the educational material and tools for people to set a behavioural goal, but we also include a chat forum to promote peer support. Moreover, additional support is offered to the participants to address low digital literacy. Physical activity is also tailored to participants' needs, accommodating any personal pain, co‐morbidities or mobility limitations. Additionally, a concurrent refinement exercise will run parallel to the intervention wherein participants' experiences will be surveyed after partaking in each component of the intervention and survey responses will be used to make refinements. Adherence and retention, as longer term outcomes, will not form part of the initial stop‐go feasibility criteria.

### 6.3 Process evaluation

A mixed‐methods process evaluation, based on the RE‐AIM framework,^21^ will be carried out to investigate individual experiences with the intervention, potential barriers and facilitators to intervention delivery, attendance (including delineation of these factors based on each different element of the intervention), and intervention fidelity.

## 7 Statistical analysis

A full statistical plan will be finalised prior to database lock before any unblinded data has been seen by the statistician. The primary analysis will compare the primary outcome (extended MACE at 24 months) between treatment groups using a Cox proportional hazards regression model, assuming assumptions are met. The model will be fitted with time to extended MACE as the outcome and treatment group as the main explanatory variable. The stratification factors (site, age, sex) will be adjusted for, and participants lost to follow‐up will be censored at the last date at which they were known to be event‐free. If any baseline imbalances become evident, these will be investigated using secondary exploratory analyses.

For the event outcomes (renal end points, extended MACE components and all‐cause mortality, amputation), the analysis approach will be a Cox proportional hazards model adjusted for the stratification factors in line with the primary analysis. The questionnaire summary measures, biomedical markers and anthropometric measures will be compared between treatment groups using linear mixed regression models adjusted for time (baseline, 12 and 24 months) and the stratification factors. Missing data for non‐event outcomes will be imputed using multiple imputation where data can be assumed to be missing at random. A sensitivity analysis will be carried out removing any variables with >10% missing data.

Summary measures (number of events or mean change from baseline) by treatment group, effect size, 95% confidence interval and two‐sided *p*‐value will be presented for each of the primary and secondary analyses. Statistical significance will be assessed at the 5% level.

## 8 DISCUSSION

Considering the prevalence of DFUD, the mortality due to CVD in people with T2D and DFUD, the resulting financial burden on the NHS,^1^, ^22^ and the limited evidence on the topic, this study is important to investigate CVD‐risk modification in this extremely at‐risk population. Notwithstanding that CVD is the leading cause of death in people with DFUD,^9^ ulcer management and/or prevention, but not CVD risks,^12^ continue to be the main focus of research and routine care^10^ in people with DFUD. While extant literature supports physical activity and exercise for blood glucose management in people living with diabetes,^23^ patients with DFUD are often told to limit weight‐bearing activity due to concerns of increased risk of foot ulcers and amputation.^24^

A Delphi study generated recommendations for physical activity and exercise in patients with diabetes at risk of ulceration.^25^ Moreover, the International Working Group on the Diabetic Foot (IWGDF) guidelines recommend that an ideal intervention for the DFUD population will consist of adequate foot ulcer offloading and increased activity levels that promote both effective ulcer healing and provide an improvement in cardiovascular health and quality of life,^26^ while ensuring participant safety. The MiFoot RCT aligns with both and has been developed to consider participants' clinical and mental health, and the SINBAD score if ulceration is active, to determine the physical activity intensity and weight bearing capacity of participants, thus providing a safe pathway for participants to exercise.

Diabetes self management education (SME) programmes are efficacious and cost‐effective in promoting and facilitating diabetes self management knowledge and skills acquisition. The 1:1 component creates an avenue for this cohort to have extra care tailored to their current health, while the social interaction during the group session component promises to address diabetes distress.^27^

While translating the positive results of any study into usual practice might be challenging, it is noteworthy that the translation of diabetes SME and cardiac rehabilitation into clinical practice and the need for these services only became established following research evidence of their beneficial effects. Upon evidenced need, therefore, it is possible to develop a new service or adapt current services to accommodate the incorporation of the MiFoot intervention. For example, current cardiac rehabilitation programmes can include physical activity tailored towards DFUD patients while the MiFoot SME classes can run concurrently with CR education.

Findings from the MiFoot RCT will provide insight into how to better treat and manage T2D‐DFUD. This potential management promises all‐encompassing benefits that covers the clinical, mental, physical and social health engagements of this cohort, which may result in a better quality of life, reduced mortality due to CVD risks and a reduced financial burden on the NHS.

## AUTHOR CONTRIBUTIONS

TO, PH and MF prepared the manuscript. KK and MD developed the study idea. PH, AB, CG, DP, DB, DW, EG, FG, FZ, AG, JP, JV, LG, MD, MH, ST and KK supported the development of the trial design. MC, VH, VJ, HR, DB and MH developed the MiFoot intervention, supported by KK, HP, FG and DW. All authors reviewed and approved this manuscript.

## FUNDING INFORMATION

This project is funded by the National Institute for Health and Care Research (NIHR) under its Programme Grants for Applied Research Programme and Diabetes UK (NIHR202021). The views expressed in this publication are those of the author(s) and not necessarily those of the NIHR, NHS or the Department of Health and Social Care.

## CONFLICT OF INTEREST STATEMENT

KK has acted as a consultant, speaker or received grants for investigator‐initiated studies for Astra Zeneca, Bayer, Novartis, Novo Nordisk, Sanofi‐Aventis, Lilly and Merck Sharp and Dohme, Boehringer Ingelheim, Oramed Pharmaceuticals, Pfizer, Roche, Daiichi‐Sankyo and Applied Therapeutics. FZ: consultancy or speaker for Menarini, Daiichi Sankyo, Servier. JV was the National Clinical Director for diabetes and obesity at NHS England from April 2013 to September 2023 and is currently the National Clinical Lead for Multiple Long‐Term Conditions at NHS England. JV is supported by the North West London NIHR Applied Research Collaboration and the Imperial NIHR Biomedical Research Centre; and he receives support from CW+, the official charity of Chelsea and Westminster Hospital NHS Foundation Trust. FG is the Clinical Lead for the National Diabetes Foot Care Audit of England and Wales. ST has acted as a consultant, speaker or received grants for investigator‐initiated studies for Astra Zeneca, Bayer, Novo Nordisk, Merc, Grunenthal, Viatris, Berlin‐Chemie, P&G, Worwag Pharma, Medtronic, Nevro, Angelini, NeuroPN, Confo‐Therapeutics, Merz and Withings. FZ has acted as a consultant and received a consultancy fee from Menarini, Servier and Daiichi Sankyo. The views expressed in this publication are those of the author(s) and not necessarily those of the NIHR, NIH, NHS or the UK Department of Health and Social Care.
